# Comparative venom gland transcriptome analysis of the scorpion *Lychas mucronatus *reveals intraspecific toxic gene diversity and new venomous components

**DOI:** 10.1186/1471-2164-11-452

**Published:** 2010-07-28

**Authors:** Zhao Ruiming, Ma Yibao, He Yawen, Di Zhiyong, Wu Yingliang, Cao Zhijian, Li Wenxin

**Affiliations:** 1 State Key Laboratory of Virology, College of Life Sciences, Wuhan University, Wuhan, 430072, People's Republic of China

## Abstract

**Background:**

*Lychas mucronatus *is one scorpion species widely distributed in Southeast Asia and southern China. Anything is hardly known about its venom components, despite the fact that it can often cause human accidents. In this work, we performed a venomous gland transcriptome analysis by constructing and screening the venom gland cDNA library of the scorpion *Lychas mucronatus *from Yunnan province and compared it with the previous results of Hainan-sourced *Lychas mucronatus*.

**Results:**

A total of sixteen known types of venom peptides and proteins are obtained from the venom gland cDNA library of Yunnan-sourced *Lychas mucronatus*, which greatly increase the number of currently reported scorpion venom peptides. Interestingly, we also identified nineteen atypical types of venom molecules seldom reported in scorpion species. Surprisingly, the comparative transcriptome analysis of Yunnan-sourced *Lychas mucronatus *and Hainan-sourced *Lychas mucronatus *indicated that enormous diversity and vastly abundant difference could be found in venom peptides and proteins between populations of the scorpion *Lychas mucronatus *from different geographical regions.

**Conclusions:**

This work characterizes a large number of venom molecules never identified in scorpion species. This result provides a comparative analysis of venom transcriptomes of the scorpion *Lychas mucronatus *from different geographical regions, which thoroughly reveals the fact that the venom peptides and proteins of the same scorpion species from different geographical regions are highly diversified and scorpion evolves to adapt a new environment by altering the primary structure and abundance of venom peptides and proteins.

## Background

More than 400 million years of evolution does not make the scorpions alter their morphology. However, although this one of the oldest arachnid is highly conserved in shape all along, they occupy vast territory of the world from Africa to Asia, Australia and America. Such powerful adaptability mostly owes to their highly specialized venom apparatus which consists of the vesicle holding a pair of venom glands connected to the stinger used to inject the venom[1]. The extant scorpions can be phylogenetically divided into 14 families based on a morphological cladistic analysis, among which the Buthidae is considered to be the largest and the most medically important family[2]. As a result, this enormous family has attracted the biggest scientific interest and been extensively studied. Till now, approximate 800 scorpion species have been classified into the Buthidae family. Previous study confirmed that each one of the species may typically contain more than one hundred different peptides in venom ranging in mass from 1,000-9,000 Da[3-5]. Among the venom peptides in this range, two classes are most regarded. One of them is small neurotoxic proteins that recognize ion channels and receptors in membranes of excitable cells, and thus toxic to different organisms[6,7]. The other is antimicrobial peptides which are important defensive molecules of the ancient innate immunity[8].

It is acknowledged that the Buthidae family possesses a very different venom arsenal comparing to other non-Buthidae families[9]. Moreover, even within the Buthidae family, vast abundance difference can be observed in venom compositions between genus to genus, different species within a genus and individuals within a species[10-12]. As a result, previous study estimated that approximately 150,000 distinct polypeptides presented in about 1500 known scorpion species in the world[13]. Numerous peptides in scorpion venoms are a big treasury waiting for exploitation. Proteomic and transcriptomic approaches have already helped us to draw a rough picture of the molecular diversity of the scorpion venom components[14]. To the best of our knowledge, the transcriptomic approach is more effective in getting an overview of scorpion venom. Not only because it can reflect the biological processes inside the venom gland cells, but also provides clues to the research of evolutionary path leading to scorpion toxin diversification directly[4].

**Figure 1:**
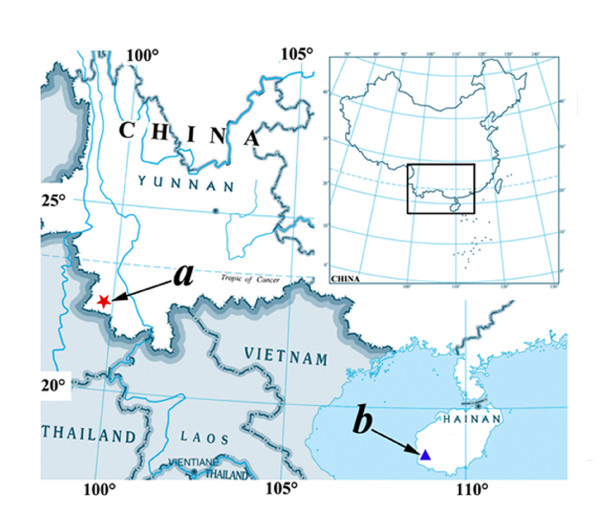
**Geographical locations of two populations of *Lychas mucronatus***. The marker (a) shows Shidian county in Yunnan province and (b) shows Janfeng mountain in Hainan island.

The family Buthidae has drawn most of attention because of its medical importance. Beyond all doubt, the venom peptides from family Buthidae will play an important role in studying biological systems and be a treasure for use in drug development. However, up to date, research interests mostly concentrate on a few genera in this family such as *Androctonus*[15], *Buthus*[16], *Mesobuthus*[17], *Parabuthus*[18], *Centruroides*[19], or *Tityus*[20]. In a sort of sense, other almost eighty genera are totally ignored, despite whose venom components are extremely attractive. *Lychas mucronatus *belonging to the genus Lychas of the family Buthidae is one of these scorpion species lacking of concern. *Lychas mucronatus *is widely distributed in Southeast Asia and southern China including Hainan island[21]. This small-sized venomous animal can be found easily in the suburbs of Yunnan, Guangxi and Hainan provinces[22]. Recently, our group collected one population of *Lychas mucronatus *from Hainan province. We performed its venom transcriptome analysis, and some new toxins were identified[23-25]. After that, we also obtained another population of the scorpion *Lychas mucronatus *from Yunnan province (Figure 1) which subsequently identified to be the same species with Hainan-sourced population according to morphological identification. It provided an opportunity to give a faithful and particular investigation on intraspecific diversity of scorpion venom peptides exist at the transcript and gene levels. Although previous researches have demonstrated that there are intraspecific molecular diversity exiting in geographic isolated scorpion populations, these researches used mtDNA phylogenetic reconstruction or limited proteomic information to elucidate how the subspecies of scorpion evolved accompanied with biogeographic events[26,27]. Comprehensive and accurate information about intraspecific variation of scorpion venom peptides and proteins is still unrevealed. In this work, we constructed a non-normalized cDNA Library and carried an EST approach to overview the transcriptome of the venom gland of the scorpion *Lychas mucronatus *from Yunnan province. A great deal of venom peptides and proteins belonging to known or atypical toxin types were identified. Most high abundance toxin types were compared with previous results conducted on Hainan-sourced population. This work provides a comprehensive comparative analysis of venom transcriptomes of two *Lychas mucronatus *populations. The results reveal the fact that both the primary structure and abundance of venom peptides are quite different in the same scorpion species from different geographical regions. Besides, transcriptomic comparison between two populations of *Lychas mucronatus *also provides some clues to understand how scorpion is reorganizing its venom toxins to adapt a new environment in evolutionary development.

## Results

### Morphological comparison of two *Lychas mucronatus *populations

*Lychas mucronatus *is one scorpion species widely distributed in Southeast Asia. In China, they chiefly exist in Yunnan, Guangxi and Hainan provinces. Our group gets two populations of the scorpion *Lychas mucronatus *successively from Hainan and Yunnan provinces with distinct ecotopes (Figure 1). Based on Kovařík and Zhu's description[21,22], the adults of *Lychas mucronatus *can be easily recognized based on coloration and morphological characteristics as following: (1) Generally, *Lychas mucronatus *is 40-65 mm long, carapace, mesosomal tergites and legs are yellow and blotched, metasoma segments are yellow to yellowish-brown from base to terminal, pedipalp, patella is predominantly dark, pedipalps manus is bright yellow with sparse, minute black spots and fingers are dark yellow brown; (2) Pedipalp fingers of males are curved, whereas those of female are straight; (3) Second segment of metasoma has 10 keels, and the third metasomal segment has 8 keels; (4) Sixth cutting edge on movable fingers of pedipalps has 3 granules; (5) Pectinal teeth number is 16-26 (frequently 19-23); (6) Metasoma of males has the same length as that of females. According to microscopic observation, both scorpion populations we collected possess the same characters mentioned above. So we convinced that two scorpion populations obtained from two provinces actually belong to the same species *Lychas mucronatus*.

### EST sequencing and clustering

**Figure 2:**
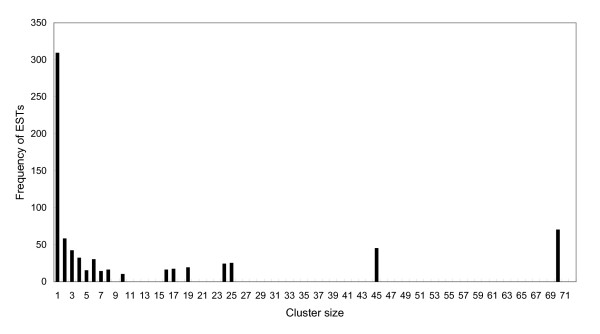
**ESTs distribution by cluster size**. For instance, there are 8 clusters of size 4, accounting for a sum of 32 ESTs.

The Yunnan-sourced *Lychas mucronatus *cDNA library was constructed with the same methods as Hainan-sourced *Lychas mucronatus*[23]. Both of libraries are not amplified, their clone number rates of different types of venom peptides and proteins should reflect the actual transcriptive abundance in original biological samples. The Yunnan-sourced *Lychas mucronatus *cDNA library possessed a 2.6 × 10^6 ^cfu/ml titer with more than 99% recombinant efficiency. In order to get more types of peptides and proteins, we selected 1000 random colonies for sequencing which have a total of 738 readable sequences. These EST sequences were then submitted into the dbEST (accession numbers: GT028570-GT029307). After processed by EGassembler online bioinformatics service[28], 738 ESTs are assembled as 309 singletons and 71 contigs consisting of two or more ESTs (Figure 2).

**Figure 3:**
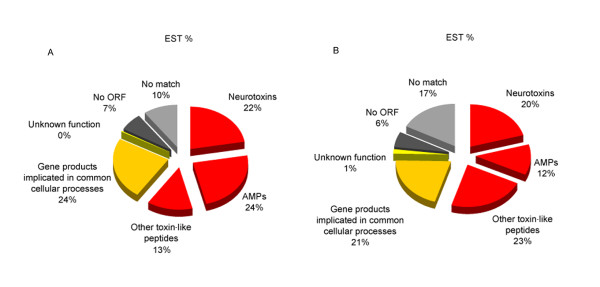
**Relative proportion of each category of total transcripts from the venom gland library of two *Lychas mucronatus *populations**. "Neurotoxins" includes transcripts encoding putative toxins specific for potassium channels from α and β-families, toxins specific for sodium channels and calcines against ryanodine receptors. "AMPs" includes Scamp, Ponericin-like peptides, bpp like, Glycine-rich peptides, and Anionic peptides. "Other toxin-like peptides" includes LVPs, PLA2, La1-like peptides, SPSV and so on. "Gene products implicated in common cellular processes" includes transcripts encoding for proteins involved in cellular processes. "Unknown function" includes ESTs similar with already described sequences with no functional assessment. "No ORF" includes sequences with non-identified open reading frame. "No match" includes ESTs having no homologous sequences in GenBank NCBI database. (A) Relative proportion of each category of total transcripts from the venom gland library of Hainan-sourced *Lychas mucronatus*. (B) Relative proportion of each category of total transcripts from the venom gland library of Yunnan-sourced *Lychas mucronatus*.

We compared the consensual cluster sequences against SWISS-PROT and GenBank NCBI databases by BLAST algorithms for a functional classification of these unique sequences. 189 clusters (488 ESTs) showed homology to existent peptides or proteins (Expect value <e-4), whereas the other 191 clusters (250 ESTs) hadn't good matches (Table 1). Among these "match" sequences, 83 clusters (414 ESTs) are deduced to be secretory peptides and proteins. These putative toxins and secretory venom components represent nearly 56% of total ESTs. It is worth noticing that neurotoxins account for 20% of the whole transcripts and peptides putative to have the antimicrobial function account for 12%. For those "non-match" ESTs, the longest ORFs were generally predicted and screened for possible signal peptides. The result indicated that 25 clusters (79 ESTs) are supposed to possess a signal peptide. This part of non-matched clones obviously a possible source for new venom peptides. 127 clusters (128 ESTs) don't have signal peptide and 39 clusters (43 ESTs) haven't ORF found. The total unassigned ESTs account for 33% which is similar to the results of other transcriptome studies[4,29]. Particular information about bioinformatical analysis of Hainan-sourced *Lychas mucronatus *cDNA library can be got from our submitted work "Evolution of the scorpion venom arsenal inferred by comparative transcriptome and molecular phylogeny analyses" (unpublished data). The distribution of all ESTs in both populations was depicted in Figure 3.

**Table 1:** Distribution of 380 clusters assembled from the scorpion *Lychas mucronatus *collected from Yunnan province

Category	Secretory (clusters/ESTs)	Non-secretory (clusters/ESTs)	Non ORF (clusters/ESTs)
Matching sequences			

Similar to venom peptide transcripts	50(325)		

Not similar to venom peptide transcripts	8(10)	131(153)	

Non-matching sequences	25(79)	127(128)	39(43)

			

Total	83(414)	258(281)	39(43)

### Known toxin types

The "-ome" approaches have already helped to reveal a great number of scorpion venom toxin types[4,10,30]. Most of these molecules are named as known toxin types because they have been identified for function or at least their primary structures were reported in literature[3,31,32]. However, there always have new scorpion venom toxins with special primary structures and unrevealed functions been reported continuously[33]. This part of toxins is named as atypical toxin types. From the venom gland cDNA library of the scorpion *Lychas mucronatus *collected from Yunnan province, sixteen known toxin types were obtained. They are encoded by 342 ESTs (57 clusters), accounting for approximately 46% of the total venom gland transcripts. All these sequences possess signal peptides. Most of these types have been identified at the protein level, whereas a few of them are only found at the transcript level. The particular comparison of these known toxin types from Yunnan *Lychas mucronatus *and previously identified known toxin types from Hainan *Lychas mucronatus *will be detailed in subjects below (Table 2).

**Table 2:** Comparison of abundance of different toxin types between two populations of *Lychas mucronatus*

Toxin types	Scorpion population			
	**Yunnan-sourced**		**Hainan-sourced**	

	**ESTs**	**clusters**	**ESTs**	**clusters**

NaTx	43	12	53	16

α-KTx	94	11	43	8

β-KTx	12	4	24	2

LVPs	69	7	10	6

calcine	1	1	2	2

scamp	26	2	30	2

plamp	9	1	15	1

bpp like	24	1	32	2

anionic peptide	25	2	43	3

Glycine-rich peptide	2	2	11	4

SPSV	4	2	2	2

PLA 2	1	1	1	1

8C-toxin	4	2	3	3

salivary protein	11	4	7	3

La1-like peptide	3	2	2	1

Metalloprotease	13	2	1	1

TIL peptide	0	0	1	1

Cytotoxic peptide	0	0	1	1

ClTx	0	0	1	1

Atypical possible toxin types	62	19	44	23

Total Toxin-like peptides	403	75	326	83

#### NaTx (toxins specific for sodium channels)

Till now, NaTx were only found in the venom of the family Buthidae except one peptide phaiodotoxin from the family Iuridae[34]. They are 6500-8500 Da (58-76 residues) polypeptides showing the conserved structure core constrained by three disulfide bridges: two disulfides link the α-helix to the anti-parallel β-sheet and the third disulfide links the β-sheet to an extended segment preceding the helix. But the fourth disulfide bridge, also called wrapper disulfide bridge[35], varies in position, and sometimes is even lost. These structure-conserved 'long-chain' peptides are considered to be the primary causes of the neurotoxic symptoms induced by scorpion envenomation.

We obtained 43 NaTxs transcripts from the venom gland cDNA library of Yunnan-sourced *Lychas mucronatus*, accounting for nearly 11% of whole toxin-like peptide sequences. These sequences were grouped in 12 clusters. According to sequence similarity and targeting receptor site, NaTxs are assorted into two patterns: α-NaTxs and β-NaTxs[36]. 6 clusters putative α-NaTxs encoded by 11 ESTs were found in Yunnan-sourced population, whereas putative β-NaTxs possess of an obviously higher abundance which have 32 ESTs encoding 6 clusters.

One cluster of putative β-NaTxs (GT028621 and GT028626) encoded by 16 transcripts is the most abundant NaTx cluster in Yunnan-sourced population library. This cluster contains two almost identical sequences differing by only two amino acids. The transcript GT028621 encodes a mature peptide of 63 amino acid residues with 8 cysteines which would form a disulfide bridges pattern of "C-C-CX_3_C-C-CXC-C" (C, cysteine; X, amino acids of various types). This peptide shows homology to the beta-toxin Toxin CsEv1 identified from *Centruroides sculpturatus*. This homology may indicate that the most abundant 'long-chain' neurotoxin in the venom of Yunnan-sourced *Lychas mucronatus *can affect sodium channel activation by binding voltage-independently at site-4 of sodium channels. Although the most abundant NaTx transcripts (EU159276 and EU159292) from Hainan-sourced library have the same cysteine pattern as GT028621, obvious diversity can be observed in primary structures. Specially, GT028621 don't contain a basic amino acid residue at C-terminal region, which shows that C-terminal modification would not exist at the post-translational level.

**Figure 4:**
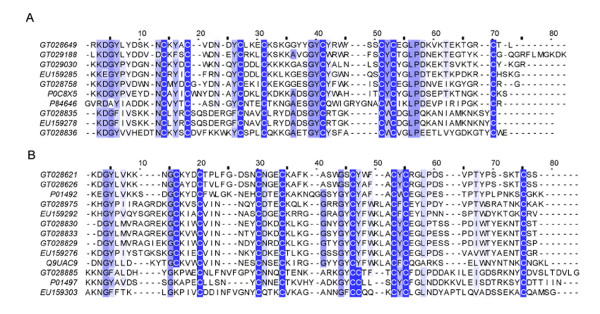
**Sequence alignment of NaTx**. GT02s represent the dbEST accession numbers for Yunnan-sourced *Lychas mucronatus *cDNA library. EU15s represent the GenBank accession numbers for Hainan-sourced *Lychas mucronatus *cDNA library. (A) Sequence alignment of α-NaTx. P0C8X5 represents Toxin Tst3 from *Tityus stigmurus*, P84646 represents Alpha-toxin OD1 from *Odontobuthus doriae*. (B) Sequence alignment of β-NaTx. P01492 represents Toxin CsEv1 from *Centruroides sculpturatus*, Q9UAC9 represents BmKAS from *Mesobuthus martensii*, P01497 represents AaH IT1 from *Androctonus australis*.

Although no significant difference was observed in the NaTx types from two scorpion populations of *Lychas mucronatus*, the NaTxs were distinct in primary structures (Figure 4). Moreover, the abundance of NaTxs was quite different. NaTxs account for nearly 16% of all toxin-like peptides in Hainan-sourced population, but 11% in Yunnan-sourced population. In addition, both scorpion populations possessed of a high abundant of putative β-NaTxs.

#### α-KTx (α subfamily of toxins specific for potassium channels)

α-KTx is widely spread in all scorpion species ever studied. This main toxin type possesses from 25 to 45 amino acid residues well packed with three or four disulfide bridges[31]. Their conserved three-dimensional structures are always constituted by a α-helix connected to a two or three-stranded β-sheet[37].

**Figure 5:**
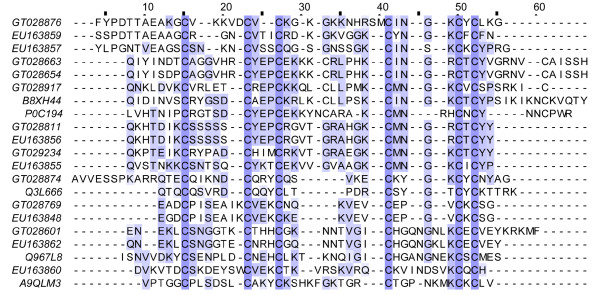
**Sequence alignment of α-KTxs**. GT02s represent the dbEST accession numbers for Yunnan-sourced *Lychas mucronatus *cDNA library. EU15s represent the GenBank accession numbers for Hainan-sourced *Lychas mucronatus *cDNA library. A9QLM3 represents the first reported toxin (LmKTx8) from Hainan-sourced population. Q967L8 represents the defensin-like protein TXKS2 from *Mesobuthus martensii*. B8XH44 represents the putative potassium channel toxin Tx771 from *Buthus occitanus Israelis*. Q3L666 represents the KTx-like peptide kk4 from *Mesobuthus martensii*. P0C194 represents the male-specific potassium channel inhibitor IsTX from *Opisthacanthus madagascariensis*.

In the scorpion *Lychas mucronatus *from Yunnan province, we obtained 11 clusters (94 ESTs) encoded for α-KTx. Among them, 9 clusters of putative α-KTxs are constrained by 3 disulfide bridges, and the other 2 clusters are packed with four disulfide bridges (Figure 5).

GT028876 represents a 3 ESTs cluster encoding a putative α-KTx. It has a mature peptide of 42 amino acid residues. Because of its unique primary structure, no homologies were shown after BLAST search. However, two clusters represented by EU163859 and EU163857 from Hainan-sourced population show some homology to GT028876. In respect that they have the same cysteine pattern as those function identified potassium channel toxins[25]. GT028876 may be a new group of short chain K^+ ^channel blockers.

GT028663 and GT028654 belong to the most abundant α-KTx cluster which is encoded by 70 ESTs. Both transcripts were supposed to encode a mature peptide with 49 amino acid residues. The mature peptides are constrained by 4 disulfide bridges, which is distinct to most other putative α-KTx toxins obtained from both scorpion populations. Blast search presented homologous Tx771 putative potassium channel toxin from *Buthus occitanus Israelis *and male-specific potassium channel inhibitor IsTX from *Opisthacanthus madagascariensis *which was proved to be a blocker to voltage-gated potassium channels Kv1.1 and Kv1.3. Surprisingly, the highest abundant toxin type from Yunnan-sourced *Lychas mucronatus *has no homologous transcript in Hainan-sourced population. Although there is still no clue on exact function of this α-KTx cluster, it is maybe very important for the survival of Yunnan-sourced *Lychas mucronatus*. Besides, another 2 ESTS cluster represented by GT028917 also possesses of the same cysteine pattern as above-mentioned sequences. These 72 ESTs form another new group of short chain K^+ ^channel blockers together. EU163848 represents the most abundant α-KTx cluster in Hainan-sourced population. It possesses of a 32 amino acid mature peptide with a pI of 4.82. This 31 ESTs encoded acidic α-KTx cluster has homologous cluster represented by GT028769 in Yunnan-sourced population. But the abundance of these acidic putative K^+ ^channel toxins is quite distinct between two populations and their functions are also to be identified.

Potassium scorpion toxins are usually classified into 4 subfamilies: α, β, γ and κ KTxs[13]. Each subfamily consists of several groups. Based on similar analysis as described before[38], GT028811 and GT029234 belong to α-KTx12 group, GT028874 belongs to α-KTx17 group, and the other short chain KTxs constitute at least 4 new groups. α-KTx is the most abundant toxin type in Yunnan-sourced population. It takes parts of nearly 23% of total toxin-like peptides, which is nearly 2-fold higher than 13% in Hainan-sourced population. In a word, not only the primary structures, but also the abundance of α-KTxs between two scorpion populations displayed high diversity.

#### Other neurotoxins

**Figure 6:**
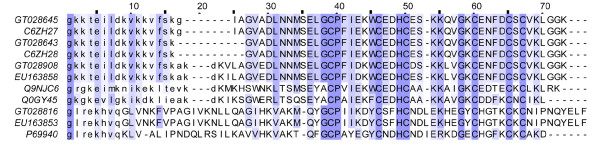
**Sequence alignment of β-KTxs**. GT02s represent the dbEST accession numbers for Yunnan-sourced *Lychas mucronatus *cDNA library. EU15s represent the GenBank accession numbers for Hainan-sourced *Lychas mucronatus *cDNA library. C6ZH27 and C6ZH28 represent LmKTx6 and LmKTx17 identified from Hainan-sourced scorpion *Lychas mucronatus*. P69940 represents the first β-KTx member TsTXK beta identified from *Tityus serrulatus*. Q9NJC6 represents BmTXKbeta from *Mesobuthus martensii*. Q0GY45 represents potassium channel toxin TtrKIK from *Tityus trivittatus*. Lower case amino acids represent putative pro-sequences.

There are two other neurotoxin types identified from Yunnan-sourced *Lychas mucronatus*. One type belongs to β-KTx, which is previously called "orphan peptides". This type is constituted by a freely moving amino acid sequence at N-terminal region and a cysteine-stabilized αβ motif at C-terminal region[39]. We obtained 4 clusters (12 ESTs) encoding β-KTx from Yunnan-sourced *Lychas mucronatus *(Figure 6). The primary structures of these long-chain K^+ ^channel toxins show high homology between two scorpion populations. It was worth mentioning that two transcripts GT028643 and GT028645 with only 3 amino acid residues difference in N-terminal region were proved to be encoded by a single gene and generated by alternative 3' splice sites. The results of electrophysiological experiments showed that the recombinant protein of these two transcripts have a very weak effect to block voltage-gated potassium channels Kv1.1 which means the real target of these β-KTxs is still waiting to be identified (Data preparing for publish). They also represent the most abundant cluster of β-KTx in the venom of Yunnan-sourced *Lychas mucronatus*. Based on similar analysis as described before[13,38], GT028816 is a new member of β-KTx1 group, while GT028643, GT028645 and GT028908 are new members of β-KTx2 group. All the ESTs encoded for β-KTx in Yunnan-sourced population take parts of 3% of whole toxin-like peptides which is obviously less than the 7% in Hainan-sourced population.

**Figure 7:**
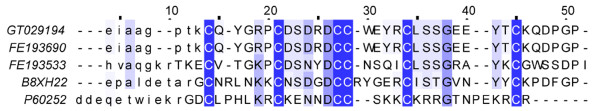
**Sequence alignment of Calcine**. GT02s represent the dbEST accession numbers for Yunnan-sourced *Lychas mucronatus *cDNA library. FE19s represent the dbEST accession numbers for Hainan-sourced *Lychas mucronatus *cDNA library. B8XH22 represents Tx758 from *Buthus occitanus Israelis*. P60252 represents Opicalcine-1 from *Opistophthalmus carinatus*. Lower case amino acids represent putative pro-sequences.

Till now, fewer Ca^2+ ^channel toxins have been obtained from scorpion venoms[40]. This type of toxin specific for ryanodine receptors possesses of an ICK (inhibitory cysteine knot) motif at C-terminal region. One EST GT029194 encoding calcine was identified from the library of Yunnan-sourced *Lychas mucronatus*. GT029194 has an 8 amino acid residues long pro-peptide and a mature peptide of 34 amino acid residues constrained by three disulfide bridges. FE193690 from Hainan-sourced population has the same primary structure as GT029194, which suggests that the calcine is conserved between two scorpion populations (Figure 7). Both of the transcripts show the conserved cysteine pattern like Tx758 from *Buthus occitanus Israelis *and Opicalcine-1 from *Opistophthalmus carinatus*. So that, GT029194 should be a new member of calcium channel toxins group 1 based on similar analysis as described before[13,38].

#### AMPs (antimicrobial peptides)

AMPs are a wide class of venom peptides with antimicrobial functions[8]. It is an important defensive weapon of innate immunity for scorpion[41]. AMPs take on great diversity in primary amino acid sequences. Most of them are short cationic peptides[42], and are structurally divided into three groups, namely linear peptides with an amphipathic α-helix, cysteine-rich peptides with one or several disulfide bridges[43], and peptides with a predominance of specific amino acids such as glycine, proline and histidine[44]. In a word, AMPs form the first line of host defense against pathogenic infections.

In Yunnan-sourced *Lychas mucronatus*, 5 type peptides are presumed to belong to AMPs: Short cationic antimicrobial peptides (Scamp), Ponericin-like antimicrobial peptides (Plamp), bradykinin-potentiating peptide like (bpp like), Glycine-rich peptides and Anionic peptides.

**Figure 8:**
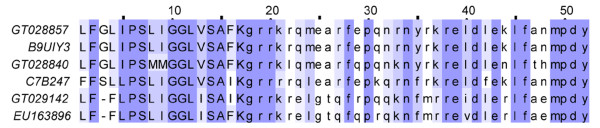
**Sequence alignment of Scamp**. GT02s represent the dbEST accession numbers for Yunnan-sourced *Lychas mucronatus *cDNA library. EU15s represent the GenBank accession numbers for Hainan-sourced *Lychas mucronatus *cDNA library. Lower case amino acids represent putative pro-sequences. B9UIY3 represents Mucroporin identified from Hainan-sourced population, and C7B247 represents Imcroporin from *Isometrus maculates*.

Scamp is a type of low molecular weight peptides belonging to the family of cationic host defense peptides[45]. This toxic type shows homology to small linear cationic toxins BmKb1 identified from the scorpion *Mesobuthus martensii*[46]. This kind of peptide always possesses of a conserved proline in the seventh amino acid residue and a lysine in the C-terminal region of mature peptide[24]. Our group had already identified one Scamp mucroporin from the Hainan-sourced *Lychas mucronatus*, which can effectively inhibit standard bacteria, especially gram-positive bacteria. From Yunnan-sourced *Lychas mucronatus*, we obtained 2 clusters (26 ESTs) of Scamp. Among them, GT028857 shared the same primary structure as mucroporin. Actually, both two clusters of Scamp obtained from Yunnan-sourced *Lychas mucronatus *have homologous transcripts in Hainan-sourced population venom (Figure 8). Scamp possesses of a high abundance in both scorpion populations venom (more than 6% in Yunnan-sourced population and 9% in Hainan-sourced population).

**Figure 9:**
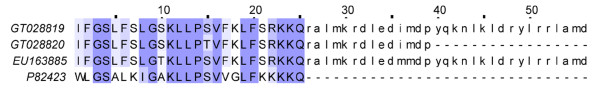
**Sequence alignment of Ponericin-like peptides**. GT02s represent the dbEST accession numbers for Yunnan-sourced *Lychas mucronatus *cDNA library. EU15s represent the GenBank accession numbers for Hainan-sourced *Lychas mucronatus *cDNA library. Lower case amino acids represent putative pro-sequences. P82423 represents Ponericin-W1 from *Pachycondyla goeldii *(Ponerine ant).

From Yunnan-sourced *Lychas mucronatus*, we obtained 1 clusters (9 ESTs) encoded for Plamp, which was a homologous toxin type of Ponericins identified from ant venom with a high activeness against Gram-positive and Gram-negative bacteria[47]. Plamp has a mature peptide consist of 24-25 amino acid residues, most of which are Lys and Arg. The primary structures of Plamps between two scorpion population are greatly conserved (Figure 9), but its abundance in Yunnan-sourced population is obviously lower than Hainan-sourced population.

**Figure 10:**
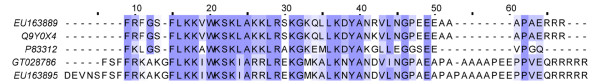
**Sequence alignment of bpp like**. GT02s represent the dbEST accession numbers for Yunnan-sourced *Lychas mucronatus *cDNA library. EU15s represent the GenBank accession numbers for Hainan-sourced *Lychas mucronatus *cDNA library. Q9Y0X4 represents Bradykinin-potentiating peptide BmK3 from *Mesobuthus martensii*, P83312 represents Parabutoporin from *Parabuthus schlechteri*.

Until now, bpp like are only characterized from the venom of family Buthidae. This type of toxin is represented by Bradykinin-potentiating peptide BmK3 identified from the scorpion *Mesobuthus martensii*[48]. From Yunnan-sourced *Lychas mucronatus*, we obtained 1 clusters (24 ESTs) encoded for this toxin type, represented by GT028786. GT028786 has almost identical primary structure like EU163895 from Hainan-sourced population (Figure 10). According to sequence alignment analysis, the N-terminal region of GT028786 shows homology to parabutoporin which has a strong antibacterial activity against Gram-negative bacteria and fungal[49]. As a result, the special primary structure of scorpion bpp like with one putative antimicrobial region at N-terminus and the other putative Bradykinin-potentiating region at C-terminus makes it to be a hypothetic bi-function protein.

**Figure 11:**
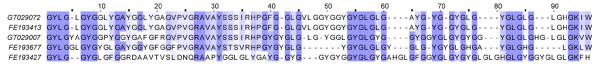
**Sequence alignment of Glycine-rich peptides**. GT02s represent the dbEST accession numbers for Yunnan-sourced *Lychas mucronatus *cDNA library. FE19s represent the dbEST accession numbers for Hainan-sourced *Lychas mucronatus *cDNA library.

Glycine-rich peptides are a type of specific amino acid predominant peptides first characterized from scorpion by our group. In this work, 2 ESTs encoded for Glycine-rich peptides were obtained from Yunnan-sourced population (Figure 11). After a multiple sequence alignment analysis, we could find that Glycine-rich peptides in Hainan-sourced population have more diversity and also a higher abundance.

**Figure 12:**
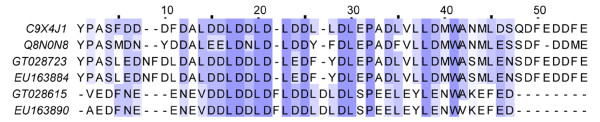
**Sequence alignment of Anionic peptides**. GT02s represent the dbEST accession numbers for Yunnan-sourced *Lychas mucronatus *cDNA library. EU15s represent the GenBank accession numbers for Hainan-sourced *Lychas mucronatus *cDNA library. C9X4J1 represents Anionic peptide from *Tityus discrepans*, Q8N0N8 represents Anionic peptide BmKa2 from *Mesobuthus martensii*.

Anionic peptides are a type of acidic venom peptides rich in aspartic acid and glutamic acid residues. Such toxin type was previously obtained from both the family Buthidae and non-Buthidae[30,50]. To the best of our knowledge, this acidic toxin type takes a higher abundance in venom of family Buthidae than non-Buthidae. Considering the family Buthidae possesses a higher abundance of basic neurotoxins in venom. It may imply that Anionic peptides play an important role in balancing the pH value of scorpion venom liquid[30]. 2 clusters (25 ESTs) encoded for Anionic peptides were obtained from Yunnan-sourced population. Both clusters represented by GT028615 and GT028723 have homologous transcripts in Hainan-sourced population (Figure 12). In Hainan-sourced population, anionic peptides account for 12% of total toxin-like peptides, which is 2-fold higher than 6% in Yunnan-sourced population.

Although there was still no consensus about the role of AMPs in scorpion venom, they were postulated to have the functions of protecting the scorpion from bacterial infection, depolarizing neural cells inducing immobilization of prey and potentiating the action of other neurotoxins within the venom[51]. 8 clusters (86 ESTs) encode for AMPs were obtained from Yunnan-sourced *Lychas mucronatus*, which accounted for 21% of all toxin-like peptides. However, the abundance of AMPs reached remarkable 40% in Hainan-sourced *Lychas mucronatus*. The primary structure of AMPs from Hainan-sourced *Lychas mucronatus *showed more diversity. This comparison suggested that Hainan-sourced *Lychas mucronatus *definitely can protect itself more effectively from microorganisms.

#### Other venom components

**Figure 13:**
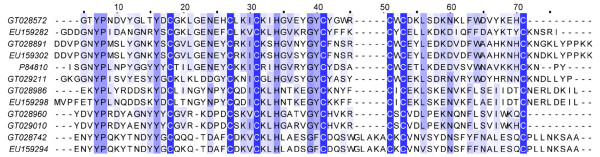
**Sequence alignment of LVPs**. GT02s represent the dbEST accession numbers for Yunnan-sourced *Lychas mucronatus *cDNA library. EU15s represent the GenBank accession numbers for Hainan-sourced *Lychas mucronatus *cDNA library. P84810 represents Lipolysis-activating peptide 1-alpha chain from *Buthus occitanus tunetanus*.

These venom components include 7 known toxin types and nineteen atypical types of venom molecules. The 7 known toxin types are lipolysis activating peptides (LVPs), phospholipase A2 (PLA2), serine protease from scorpion venom (SPSV), metalloprotease, salivary protein, La1-like peptides and 8C toxin. LVPs are a group of long chain toxins shared high homology to those NaTxs but in defect of a cysteine. This structure makes LVPs form an interchain disulfide bridge and exert distinct biological activity on adipocyte lipolysis[52]. 7 clusters (69 ESTs) encoding LVPs were identified from Yunnan-sourced *Lychas mucronatus*. It was worth mentioning that LVPs were the second most abundant toxin type in the venom of Yunnan-sourced population. It accounted for 17% of whole toxin-like peptides far more than 3% in Hainan-sourced population. Although the abundance of LVPs was quite distinct in two populations, their primary structures seemed conserved (Figure 13). PLA2, SPSV and metalloprotease are proteinases within scorpion venom. Their abundances are correspondingly lower than those "common" toxin types in venom, but without a doubt, they are very important for scorpion to prey and digest. Salivary protein is a type of high molecular weight protein in scorpion venom. Salivary protein was denominated for its homology to the salivary derived protein from tick, and this type of toxins always has a mature peptide of about 210 amino acid residues. La1 is the most abundant toxin identified from the venom of *Liocheles australasiae*[9]. But the abundance of La1-like peptides in venom of family Buthidae is not such high, and the accurate function of La1-like peptides is still unknown. 8C toxin is another new type of toxin-like peptides well packed by 4 disulfide bridges, which was first obtained by our group. Nineteen atypical types of venom molecules obtained from Yunnan-sourced population were seldom reported in other scorpion venoms. The accurate function of these atypical scorpion venom toxins is still needed to be characterized [see Additional file 1]. Other venom components accounted for nearly 41% of whole toxin-like peptides in venom of Yunnan-sourced population, which is a big natural venom resource waiting for exploring.

## Discussion

Scorpion venom is a complex mixture of biologically active peptides with diverse physiological effects[14]. Most of the peptides ever identified are disulfide-rich neurotoxins that specifically modulate various ion channels permeability of excitable and non-excitable cells[31]. However, more and more other venom components have been characterized recently, including antimicrobial peptides without disulfide bridges and other functional molecules[32,53]. In order to gain further insight into scorpion venom compositions, it is not enough to employ routine studies by protein chemistry always aiming at the isolation of specific active components. The introduction of powerful chromatographic techniques, followed by primary structure determination using automatic Edman degradation, makes it possible to produce an overview of scorpion venom compositions. Mass spectrometry was firstly used to get the mass fingerprinting of toxic fractions of *Tityus serrulatus *venom[54]. Since then, overall venom compositions of ten scorpion species have been comprehensively investigated at the proteome level, including eight species from the family Buthidae[14]. The powerful technique help to confirm the hypothesis that scorpion venom is a complex mixture of various distinct proteins with vastly abundant difference between families to families, genus to genus and different species within a genus[9]. We chose transcriptomic approach to get an overview of the Yunnan-sourced *Lychas mucronatus *venom because it's not affected by the extraction of venom and the dynamic expression of the gland or peptide maturation like proteomic approach[12]. The transcriptomic analysis is more likely to give a comprehensive comparison of the venom compositions from two *Lychas mucronatus *populations.

Some evidences already displayed the phenomenon of intraspecific diversity of scorpion venom peptides[12,26]. According to SDS-PAGE and random amplified polymorphic DNA (RAPD) techniques, the venom of *Scorpio maurus palmatus *from four geographically isolated localities in Egypt was investigated. Both obvious morphological differences and protein molecular weights diversity were observed[55]. But the particular distinctions such as primary structure and exact abundance of venom toxins among different populations of the same scorpion species are not clearly clarified. As two populations of the scorpion *Lychas mucronatus *successively obtained from Hainan and Yunnan provinces were convinced to be the same species according to morphological analysis, our study conduct on the venom components of *Lychas mucronatus *by a transcriptome approach may fitly figure out the question.

The experimental methods for constructing the venom gland cDNA library of Yunnan-sourced *Lychas mucronatus *was the same as Hainan-sourced *Lychas mucronatus*[23]. 60 adult specimens from both scorpion populations were selected when venom gland cDNA library was constructed. The gender difference of scorpions is basically half to half. We cut off scorpion venom glands 2 days after extraction of their venom by electrical stimulation. Most temporary influences such as age, seasonal, sex, feeding behavior and time for RNA transcription can be excluded. Nevertheless, the experimental results confirmed our hypothesis about the diversity exist in nearly every toxin type (Table 2).

Almost all high-abundant toxin types were obtained from two scorpion populations. Great diversity was observed in these highly expressed venom compositions, especially neurotoxins and AMPs. Among neurotoxins, short chain α-KTx was highly expressed in Yunnan-sourced population, whereas long chain NaTx and β-KTx was highly expressed in Hainan-sourced population. Particularly, α-KTx in the venom of Yunnan-sourced population is not only 2-fold higher abundant than in Hainan-sourced population, but also more diversified in primary structures. GT028663 represents the most abundant α-KTx cluster in Yunnan-sourced population, but we can not find its homologous transcripts in the venom gland cDNA library of Hainan-sourced population. EU163848 represents the most abundant α-KTx cluster from Hainan-sourced population. Although EU163848 shows homology to GT028601 from Yunnan-sourced population, EU163848 obviously has a higher expression level. Taken together, Yunnan-sourced *Lychas mucronatus *possesses a higher abundant and more diversified α-KTxs in venom and the homologous transcripts of the most abundant α-KTx cluster in the venom of Hainan-sourced *Lychas mucronatus *have a relatively low expressed level in the venom of Yunnan-sourced *Lychas mucronatus*. All these obvious diversity should be intricately related to scorpions' adaption in different environment which primarily concern the scorpions' interaction with their prey and predators. On the other hand, Hainan-sourced *Lychas mucronatus *possesses a higher abundance of NaTxs in venom. Because the NaTxs can cause severe neurotoxic symptoms after scorpion envenomation[36], Hainan-sourced *Lychas mucronatus *could be a more dangerous killer, which is more efficient for not only preying but also defensing itself from other predators.

According to the transcriptome analysis, we can conclude that both scorpion populations possess relatively high abundant AMPs. But it's unexpected that the abundance of AMPs in the venom of Hainan-sourced population reached remarkable 40% of all toxin-like peptides. Till now, more and more AMPs have been isolated and characterized[56,57]. AMPs can destroy bacteria, fungi, parasites, and even some viruses[58]. They are important defensive weapons of scorpion innate immunity. So we can assume that the high expression levels of AMPs could protect Hainan-sourced *Lychas mucronatus *away from microbial infection, and it may also implicate other unascertained benefits for the survival of the population.

Hainan island locates in Southern China and is separated from mainland by Qiongzhou strait. Because surrounded by South China Sea, the climate of Hainan island is hot and humid all the year round. Yunnan locates in southwest China and is a mountainous region, whose rainfall is relatively less than Hainan province. Hainan island is an excellent living environment for *Lychas mucronatus*. In this biotope, Hainan-sourced *Lychas mucronatus *has enough prey. But this hot and humid environment may also bring powerful nature enemy and more pathogenicbacteria. In a word, the distinctive living environments probably relate to the adaptive evolution of two *Lychas mucronatus *populations.

**Figure 14:**
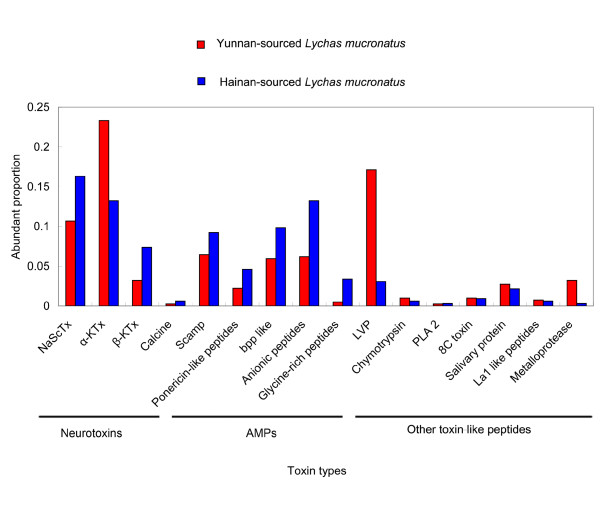
**Comparison of abundance of different toxin types between two populations of *Lychas mucronatus***. For instance, there are 94 ESTs encoded for α-KTx in the library of Yunnan-sourced *Lychas mucronatus *accounting for 24% of all the 396 ESTs encode for toxin-like peptides.

The dissimilarity of toxin-like peptides between two populations was showed in Figure 14. Although two populations of scorpion belong to the same species, diversity of venom peptides had gradually accumulated since Hainan island was separated from Chinese mainland 2.5 million years ago[59]. Long period isolation doesn't make the scorpion *Lychas mucronatus *change their toxin types much in venom, but the primary structure and abundance of these toxin types change a lot which probably related to the adaption of *Lychas mucronatus *in different environment. To the best of our knowledge, this work is the first comprehensive comparative analysis of venom transcriptomes between two populations of the same scorpion species from different geographical regions. It provides accurate difference of venom peptides between *Lychas mucronatus *from Hainan and Yunnan. Sight is no longer restricted to only protein molecular weights and concentration, but also the detailed diversity of primary structure and abundance. Besides, because two scorpion populations are obtained successively from island and mainland, *Lychas mucronatus *could represents a useful model system for studying molecular evolution and biogeography. In addition, the geographical variability in the venom of the same species also provides some clues for producing of effective anti-venoms and understanding the symptomatology of envenomation.

## Conclusions

Our work unravels a large number of venom molecules never identified in scorpion species. The result provides a comprehensive comparative analysis of venom transcriptomes of the same scorpion species from different geographical regions, which thoroughly reveals the fact that peptides and proteins of the same scorpion species from different geographical regions are highly diversified and the scorpion venom arsenal is a constantly evolving system to adapt the different biotopes. We can make a conclusion that there are far more peptides in one scorpion species venom than previous expectation considering of the geographical isolation.

## Methods

### cDNA library construction

The Yunnan-sourced *Lychas mucronatus *were collected from Shidian county of Yunnan province in September 2008. Venom glands of 60 wild specimens were cut off 2 days after extraction of their venom by electrical stimulation[60], and ground into fine powder in liquid nitrogen. Total RNA was isolated with TRIZOL Reagent (Invitrogen, Carlsbad, CA, USA), and then mRNA was prepared with PolyATtract^® ^mRNA Isolation Systems (Promega, Madison, WI, USA). SuperScript™ Plasmid System (Invitrogen) was used to construct a directional cDNA library from 6 μg mRNA. cDNA inserts were directionally cloned into the plasmids pSPORT 1 according to the supplier's instructions. The recombinant plasmids were transformed into electrocompenent cells.

### Sequencing

After growing the clones overnight in appropriate Luria Broth culture medium containing 100 μg/ml of ampicillin, random colonies were selected in order to obtain an unbiased overview of the venom gland transcriptome. After overnight culture, plasmid DNA was isolated using alkaline lysis method. Purified plasmids were single-pass sequenced on ABI 3730 automated sequencers (Applied Biosystems, Foster City, CA, USA).

### Bioinformatics analysis

Phred program were used to examine the trace files of sequenced clones, the cutoff Phred score was set to 40 as before[30,61]. Vector and adaptor sequences were removed using the program Cross Match. After removing the PolyA tail, we discarded those sequences shorter than 100 nt. High-quality sequences were processed on the website EGassembler http://egassembler.hgc.jp/ with the default parameter[28]. The resulted sequences were deposited into the dbEST. Each cluster was annotated by being searched against SWISS-PROT http://www.expasy.org/tools/blast/ and GenBank NCBI database http://www.ncbi.nlm.nih.gov/blast using BLAST algorithms with an e-value cut-off set to <10^-4^. After BLAST search, the unmatched clusters were further identified for open reading frames using the ORFfinder http://www.ncbi.nlm.nih.gov/projects/gorf/. Considering the extreme diversity of scorpion toxins, those clusters putative to encode venom peptides was re-examined manually to pick out individual different isoforms. All clusters were checked for the existence of signal peptides using the SignalP 3.0 program http://www.cbs.dtu.dk/services/SignalP/[62]. All the sequence alignment was performed by Clustal_X 1.83 software followed by manual adjustment[63], and viewed by the software Jalview[64].

## Authors' contributions

Ruiming Zhao carried out cDNA library construction, participated in the bioinformatics analysis, and drafted the manuscript. Yibao Ma participated in the alignment analysis, and drafted the manuscript. Yawen He participated in the sequencing. Zhiyong Di completed the characterization of scorpion species. Yingliang Wu participated in the design and coordination of the study. Zhijian Cao participated in the design and coordination of the study, and drafted the manuscript. Wenxin Li conceived of the study, and participated in its coordination. All authors have read and approved the final manuscript.

## Supplementary Material

Additional file 1 **Atypical venom molecules characterized from the scorpion Yunnan-sourced *Lychas mucronatus***. The data represents nineteen novel types of venom peptides with special primary structures and unrevealed functions from the scorpion Yunnan-sourced *Lychas mucronatus*. Click here for file
