# Supplementary material for: Comparative venom gland transcriptome analysis of the scorpion Lychas mucronatus reveals intraspecific toxic gene diversity and new venomous components
Source: BMC Genomics. 2010 Jul 28;11:452. doi: 10.1186/1471-2164-11-452 (PMC3091649; doi:10.1186/1471-2164-11-452)
Supplement: Additional file 1 — Atypical venom molecules characterized from the scorpion Yunnan-sourced Lychas mucronatus. The data represents nineteen novel types of venom peptides with special primary structures and unrevealed functions from the scorpion Yunnan-sourced Lychas mucronatus. [file 1471-2164-11-452-S1.DOC]

**Supplementary file**

**Table. Atypical venom molecules characterized from the scorpion Yunnan-sourced *Lychas mucronatus***

| CLUSTERS | ESTs | Sequences |
| --- | --- | --- |
| GT028582 | 17 | >GT028582  MKALCAILLVLFACSVMFEHFSISTAEKVLQNPLSELKRNCEKADCRRSLPQNKQHDFKE# |
| GT028606 | 4 | >GT028606  MRFSIISASLVLIFANVKAFNEEEILEIFCGVPKKLVSRYNQCLIDHGPEIIKKNYEIINSCMKGHLGSETESAMEYVCNKKNVDISIKRCISDKISEEMKEFDRRARLEVWDVLYVCIFKA# |
| GT028760 | 4 | >GT028760  MDIKGLLVILFFVLLITGEVENMKPKKHKYKYKNRKRFLENFEEPLQKRDMNEESYELFNQ# |
| GT028802 | 6 | >GT028802  MNTKTLIVVFLVCLLVSEVVLARRCGGGRKIKIKKIVRKLRPIVRVMKVITRMRTRRPRPRPCNSS#  >GT028805  MNTKTLIVVFLVCLLVSEVVLARRCGGGRKIKIRKIVRKLRPIVRVMKVITRMRTRRPRPRPCNSS# |
| GT028862 | 2 | >GT028862  MVSTLMIASVKLRLYCTALIILCNQKNPSEFNVLIHKLIIKWGFMNKNFHFVSVFFIKF# |
| GT028912 | 3 | >GT028912  MNFQVFSLIFFNFVYYCSCSTFEEALCELEKEERVKVLRCYEENTDPGILLYSKSFLSCLLDERASFITLNEMACGKTFSTNERRACFEEANVFLVNFTEEMKKKHKNAVKKCFDK# |
| GT029029 | 1 | >GT029029  MNKLFLFTLLVTLWSVKGFTYEEKKQAFCSLPKVYQIRLLDCLIDRGSENDKEVVNAVYKCMNEHSDVDGKADAMMKAVCNEEIFATNRNLILCMLVNPPKLEHSERTNDDDLEAVKYCLVNG# |
| GT029182 | 1 | >GT029182  MKTTLIFCILGIVIPTAVVSSQDFCGLSQQEREDYWECVERGMSADELNQAKLFLECINKQSAVELLAFFCNADSIEDSIEKEEFRLLFAECLKSHPASFNAGNESCLKEARK& |
| GT029212 | 1 | >GT029212  MIRYVLVIITCFLVAAKSHVTIGPVPNNLGFPDRSILLALVAPTCEPELEGLVDECVNNVTIRNVCYDCFREGLTKVYSYCCHKYNHMYEWCLEYFSGEMK@ |
| GT028915 | 2 | >GT028915  MNFLCILFVVSLISSLSKCTTSSMKRELDLGMSRGHSGSQVGKALLGIQSANRTDGPGRKRRSFDLYALVNAK& |
| GT028923 | 3 | >GT028923  MNSREMFCVFILFASFFYCSYAEQECNCDKSCEPVKDCTFGTAMDKCGCCEVCAVGIGHFCGKFFNNAVCAEGLKCAKLGEGADGEALEICLRA& |
| GT029162 | 1 | >GT029162  MKCLIILVIFAIATTQIRAACNQQSDCPPGSCCKKPNSYMEGGCFPLLKRGESCYVKDNRIYDVYREKCPCGEGLRCHQFAEGIWQGQCVETS# |
| GT029300 | 1 | >GT029300  MLKLVILLCVIESIYSLSCPCQWNKSLRDSCRIPENCLAGTTKDVCGCCDVCAKIEGESCGGPWNIGGSCAVGLTCVKVDKRNFHSKGVCKLN# |
| GT028580 | 2 | >GT028580  MKFSLISVFLFAVFLSNENIFQAIADNRYLPTIFKNPVNAEQRSEAVMSSSLTNEEESRNWPHRATRNTLEKGQKRSPAARSEIEEMEEYDDRWMW# |
| GT028898 | 3 | >GT028898  MKSFIIFSGLIFVIFLSNKYSCQPVADNQYLSSLSENVKVLEQRMYPAMSASSANEEKRFQLEIFGSMAKAEEQRSRYPNPLFFHSWKDTED# |
| GT028904 | 4 | >GT028904  MKFFGILLIVTMVVLVMIATTYVESISIGIKCSPSIDLCEGQCRIRKYFTGYCSGDTCHCSG&  >GT028905  MKFFGILLIVTIFVLVMIATTYVESISIGIKCSPSIDLCEGQCRIRKYFTGYCSGDTCHCSG& |
| GT028813 | 3 | >GT028813  MIIVKLFTCLLMVSSVLTLNDVRYKYYKTEKVGNKCRYNMFSYLENDESIYEDRLCKMYTCHIMEDDALMQHSMCKLPKGFREGCTVESKTGRFPYCCLNRKMSCPPEERNDDFNVVSVKGPKFPRNKSNDKFKNLSVKNCPCLE# |
| GT028808 | 1 | >GT028808  MTFITLTIGLSLRTIFLIFIFLPPPHLLARTTSLTRRQTRKTTIKVFVFILPFYQTPNW@ |
| GT028613 | 2 | >GT028613  MKSFLLIALVLFFLFVSYASAKNKCQLPSDVGKGKASFTRYYYNEEGGKCETFIYGGVGGNSNNFLTKEDCCRECAQGSC#  >GT028614  MKSFLLIALVLFFLFVSYASAKKKCQLPSDVGKGKASFTRYYYNEESGKCETFIYGGVGGNSNNFLTKEDCCRECAQGSC# |
